# Frequency Scanning Dual-Mode Asymmetric Dual-OAM-Wave Generation Base on Broadband PB Metasurface

**DOI:** 10.3390/mi13071117

**Published:** 2022-07-15

**Authors:** Jiayu Yu, Qiurong Zheng, Xueqin Tang, Jie He, Jie Liu, Bin Zhang, Kun Zou

**Affiliations:** 1 Information and Navigation College, Air Force Engineering University, Xi’an 710077, China; returntoo@163.com (J.Y.); hj1993214@163.com (J.H.); lj940107@163.com (J.L.); woodman68@163.com (B.Z.); wyyxzle@163.com (K.Z.); 2 Joint Logistics College, PLA National Defense University, Beijing 100117, China; xueqin_tang@163.com

**Keywords:** microwave passive metasurface, vortex beam, orbital angular momentum, broadband, Pancharatnam–Berry phase, frequency scanning, wave manipulation

## Abstract

Increasing information capacity is significant for high-speed communication systems in a congested radio frequency sequence. Vortex waves carrying mode orthogonal orbital angular momentum (OAM) have gained considerable attention in recent years, owing to their multiplexing quality. In this study, a broadband Pancharatnam–Berry (PB) metasurface element with a simple structure is proposed, which exhibits an efficient reflection of the co-polarized component and a full 2π phase variation in 10.5–21.5 GHz under circularly polarized wave incidence. By convolution and addition operations, the elaborate phase distribution is arranged and the corresponding metasurface-reflecting dual-mode asymmetric dual-OAM waves is constructed. Under continuous control of the working frequency, the OAM vortex beams with the topological charges 1 and −1 are steered to scan within the angle range of 11.9°–24.9° and 17.9°–39.1° at *φ* = 315° and 135° planes, respectively. The simulation and measurement results verified the feasibility of generating frequency-controlled asymmetric dual beams and the validity of dual-mode OAM characteristics, both in the near and far fields. This design approach has considerable potential in OAM wave multiplexing and wireless communication system transmission.

## 1. Introduction

Boosting information capacity is important for high-speed and -capacity wireless communication, especially in the congested radio frequency (RF) sequence. Angular momentum is one of the natural characteristics of electromagnetic (EM) waves, which consist of spin angular momentum (SAM) and orbital angular momentum (OAM) [1]. Vortex EM waves carrying OAM have hollow annular intensity profiles and a helical phase wavefront of exp(*ilθ*), where *l* is the topological charge and *θ* is the azimuthal angle. Integer *l* is theoretically infinite, and OAM beams with different modes of *l* are mutually orthogonal and independent, which offer additional degrees of freedom for EM wave spatial multiplexing and for tremendously enriching the dimensions of the communication system [2,3]. Owing to the properties of superposition and decomposition from RF to visible [4,5,6,7,8], OAM waves have reaped considerable attention and investigation in optical modulation [4,5], wireless communication [6], radar imaging [7], and other fields of research since their discovery in 1992 [9]. In the microwave domain [10,11,12,13], antenna array [11], traveling-wave antennae [12], and spiral phase reflectors [13] have been explored to generate OAM waves for information multiplexing in the hope of simultaneously improving spectrum utilization and communication transmission capabilities.

The metasurface resembles a two-dimensional artificial EM structure arranged by subwavelength elements. With a delicate modulation ability for the amplitude, phase, and polarization of EM waves, the metasurface shows great potential in new system communication [14], EM stealth [15,16,17], holography [18], and other EM applications [19,20,21,22,23,24,25,26]. It not only displays the advantages of small weight, a low profile, and easy fabrication, but also builds the feasibility for the mode multiplexing of OAM beams. Recently, metasurfaces have been reported to engender microwave vortex beams to reduce the complexity of the typical method for design and fabrication [27,28]. Through varying the structural parameters of the elements to manipulate the desired phase of the scattering field, plentiful OAM beam generators have been proposed under the irradiation of linearly polarized waves [28,29,30]. However, this approach makes it difficult to achieve the intended function within the broadband. This limitation has been overcome by the introduction of the Pancharatnam–Berry (PB) phase theory, where the local phase response is twice the spatial rotation angle in the meta-atom plane with a circularly polarized (CP) wave incidence, and without geometrical size modification. Consequently, many PB metasurfaces have been configurated to create vortex waves in the microwave domain, and they enjoy wideband and high-efficiency characteristics [31,32,33].

In the OAM wave generation studies mentioned above, most beams were aimed at a normal direction or generated the same wave mode. Because of spatial isolation, multiple beams could provide multiplexing channels for data transmission. Using cross-polarized incidence [34], aperture field superposition [35], and a metasurface array [36], several efforts have been applied to create metasurface-enabled multi-mode multiple OAM beams. In addition, a convolution operation [37] on coding metasurfaces for scattering field transform has been recently proposed by Liu, which can deflect the beam to an arbitrary direction. Combined with Wu’s phase addition theory [38], multifunction beamforming can be concurrently achieved. Following this route, multibeam multimode OAM wave generators can be acquired with improved flexibility and reduced design difficulty, further elevating the OAM space-division multiplexing capability.

**Figure 1:**
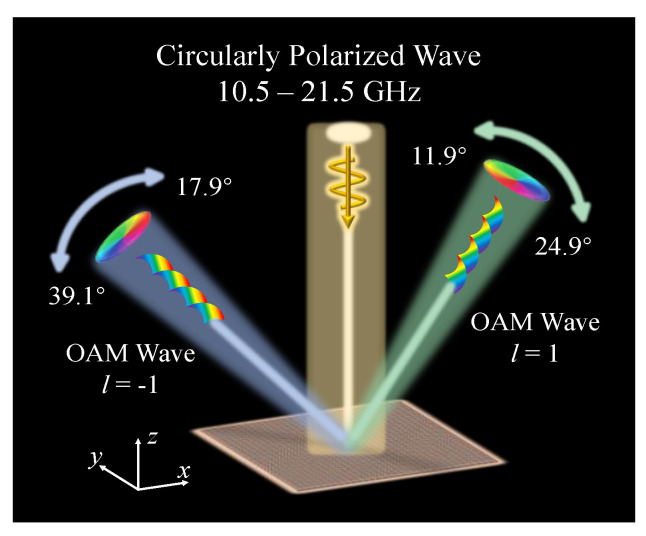
Schematic diagram of the proposed frequency scanning dual-mode asymmetric dual-OAM-wave metasurface.

In this paper, we present a single-layer wideband PB metasurface. Under CP plane-wave incidence, its co-polarized reflection coefficient exceeded 0.95, and a 0–2π continuous phase change was obtained in 10.5–21.5 GHz (68.8%). Further, 32 × 32 meta-atoms were arranged according to the tailored phase distribution to generate OAM waves with topological charges of 1 and −1, where the two asymmetric vortex beams were steered to *φ* = 315° and 135° planes, and were scanned at the angle ranges of 11.9°–24.9° and 17.9°–39.1° manipulated by frequencies, respectively. The schematic diagram of the proposed frequency-scanning metasurface is shown in Figure 1. The simulations and experiments revealed that the frequency-controlled dual-mode dual-OAM beams had good performance at both near and far fields. This proposed design has great potential to enhance the capability of OAM wave multiplexing and wireless communication system transmission.

## 2. Design and Method

### 2.1. Design of the Broadband PB Meta-Atom

**Figure 2:**
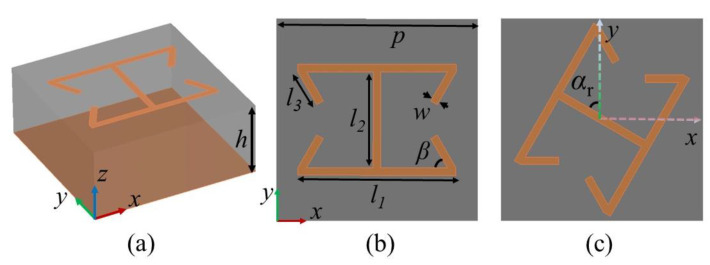
PB metasurface unit cell. (**a**) Perspective diagram. (**b**) Front view. (**c**) Rotating patch.

Figure 2a,b display the schematic and front view of the suggested PB metasurface unit cell, which includes a dielectric substrate in the middle, metallic resonator printed on the top, and reflective ground backed by the bottom. The dielectric substrate was F4B (ε_r_ = 2.2 and tanδ = 0.0009), the thickness of which was *h* = 3.4 mm. The meta-atom had a period of *p* = 8 mm, and the other optimized structural sizes are listed as *l_1_* = 6.3 mm, *l_2_* = 3.8 mm, *l_3_* = 1.5 mm, *w* = 0.3 mm, and *β* = 60°. With the metallic patch rotating angle of α_r_, the entire 2π geometric phase regulation was performed with high efficiency under the illumination of CP plane waves.

It was practical to utilize the reflected Jones matrix to evaluate the incident and scattered fields for a rotated anisotropic meta-atom. For the CP wave illumination, the reflection coefficients can be explicated as [32,39]

(1) $$R_{CP}=\left[\begin{array}{c} \begin{array}{cc} r_{--} & r_{-+} \end{array}\\ \begin{array}{cc} r_{+-} & r_{++} \end{array} \end{array}\right]=\frac{1}{2}\left[\begin{array}{c} \begin{array}{cc} (r_{xx}-r_{yy})e^{-j2\alpha }+j(r_{xy}+r_{yx})e^{-j2\alpha } & \left(r_{xx}+r_{yy})+j(r_{xy}-r_{yx}\right) \end{array}\\ \begin{array}{cc} \left(r_{xx}+r_{yy})-j(r_{xy}-r_{yx}\right) & (r_{xx}-r_{yy})e^{j2\alpha }-j(r_{xy}+r_{yx})e^{j2\alpha } \end{array} \end{array}\right]$$

where r*_xx_*, r*_yy_*, r_−−_, and r_++_ represent the co-polarized reflection coefficients of EM propagation under *x*-polarized, *y*-polarized, left-hand circularly polarized (LHCP, −), and right-hand circularly polarized (RHCP, +) normal incidences, respectively, while r*_yx_*, r*_xy_*, r_+−_, and r_−+_ are the interrelated cross-polarized reflection coefficients. r_−−_ and r_++_ hold the Pancharatnam–Berry (PB) phase, which is numerically twice the spin angle. An abrupt phase variation of e^−j2α^ (e^j2α^) from the main diagonal elements was introduced by rotating the meta-atom with an angle of α_r_.

**Figure 3:**
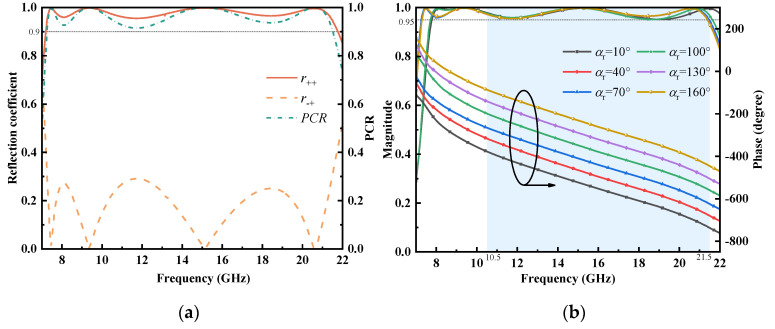
Simulation results of the proposed PB meta-atom. (**a**) Reflection coefficient and PCR of r_++_. (**b**) The magnitude and phase of r_++_ at different rotating angles.

The numerical simulation of the presented meta-atom was implemented with CST Microwave Studio with unit cell boundary conditions. Figure 3a shows that the amplitudes of co-polarized reflection coefficients r_++_ remained greater than 0.95 across the broad frequency band of 7.5–21.5 GHz, whereas the cross-polarized ones were restrained. However, for RHCP incidence, the polarization conversion rate (PCR) of the co-polarization reflection revealed polarization conversion efficiency, and it can be written as *PCR* = r_++_^2^/(r_++_^2^ + r_−+_^2^). As shown in Figure 3a, the magnitude of the PCR in the wide band was always higher than 0.9, indicating that the unit cell had a high-efficiency conversion ability. In the shaded area of Figure 3b, for various rotation angles, the phase responses kept parallel, as expected, and the co-polarized reflection amplitudes all exceeded 0.95, where the relative phase change was precisely twice the rotating degree. Therefore, the range of 10.5–21.5 GHz (68.8%) was determined to be the effective operating band, which makes the meta-atom an outstanding candidate for the assembled metasurface with high-performance phase modulation features.

### 2.2. Design of Frequency Scanning Dual-Mode OAM Vortex Beams

Based on the suggested PB meta-atom, the deflected dual-mode OAM vortex beams were visualized in a wide frequency band via an elaborately designed phase distribution.

**Figure 4:**
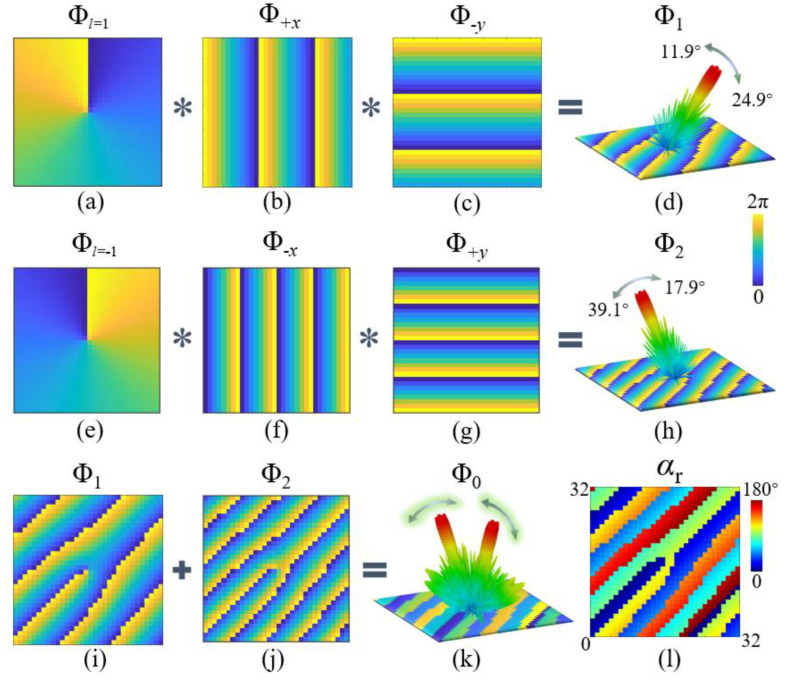
Phase distribution and rotating angles. (**a**) Phase profile Φ*_l_*_= 1_ of OAM vortex generation with topological charge *l* = 1. Phase gradient sequences (**b**) Φ +*x* and (**c**) Φ −*y* orientating to +*x*- and −*y*-directions with period Γ = 96 mm. (**c**) Phase profile Φ_1_ by the convolution operation. (**d**) Phase profile Φ_1_ by the convolution operation. (**e**) Phase profile Φ*_l_*_= −1_ of OAM vortex generation with topological charge *l* = −1. Phase gradient sequences (**f**) Φ −*x* and (**g**) Φ +*y* orientating to −*x* and +*y* directions with period Γ = 64 mm. (**h**) Phase profile Φ_2_ by the convolution operation. (**i**) Φ_1_. (**j**) Φ_2_. (**k**) Phase profile Φ_0_ by complex addition of Φ_1_ and Φ_2_. (**l**) Rotating angles α_r_ of the metallic patch.

First, the vortex beam function was generated by establishing a spiral phase profile, and the desired phase shift of each element position (*x*, *y*) needed to satisfy the relationship as

(2) $$\Phi_{l}\left(x,y\right)=l\cdot \arctan\left(\frac{y}{x}\right)$$

where *l* is the topological charge. As Figure 4a,e show, the phase profiles of *l* = 1 and −1 had opposite clock directions.

Second, following the generalized Snell’s law, the reflected deviation angle *θ* from the +*z*-axis for the EM wave normal incident was [40]

(3) $$\theta =\arcsin\left(\frac{\lambda }{\Gamma }\right)=\arcsin\left(\frac{2\pi c}{f\cdot \Gamma }\right)$$

where *f* is the frequency of the EM wave and *λ* is the corresponding wavelength in free space. Γ denotes the period of a constant phase gradient sequence. Once the period length was fixed, the deflection angle was interpreted as being related to the operating frequency. This is the key to the beam deviation with frequency in this study.

In addition, the electric field distribution of the sequence pattern on the phase modulation metasurface and the scattering mode in the far field were Fourier transform pairs; therefore, the scattering mode displacement principle can be described with the following convolution operation found in [37,41]:

(4) $$F\left(x_{\lambda }\right)\cdot e^{jx_{\lambda }\sin\theta_{0}}\overset{FFT}{\Leftrightarrow }F\left(\sin\theta \right)*\delta \left(\sin\theta -\sin\theta_{0}\right)=F\left(\sin\theta -\sin\theta_{0}\right)$$

where *F*(*x_λ_*) is an arbitrary sequence pattern, e^jxλsin*θ*^^0^ describes a periodic discontinuous phase gradient along a certain direction, and F(sin*θ* − sin*θ*_0_) can be explained as the original scattering pattern of F(sin*θ*) deviating to sinθ_0_. When two orthogonal periodic phase gradient sequences were subjected to the operating mechanism, the elevation angle *θ*_r_ and azimuth angle *φ*_r_ were calculated as

(5) $$\theta_{r}=\arcsin\left(\sqrt{\sin^{2}\theta_{1}\pm \sin^{2}\theta_{2}}\right)$$

(6) $$\varphi_{r}=\arctan\left(\sin\theta_{2}/\sin\theta_{1}\right)$$

in which *θ*_1_ and *θ*_2_ are the deviation angles along the *x*- and *y*-axes, respectively. In Figure 4b,c, constant phase gradient sequences orient to +*x*- and -*y*-directions, the period Γ_1_ of which were both determined to be 96 mm. After the convolution operation of the three phase profiles in the first row of Figure 4, the OAM vortex wave with the topological charge *l* = 1 was deflected to azimuth *φ*_r1_ = 315°. The beam scanned within the elevation angle *θ*_r1_ range of 11.9°–24.9°, controlled by the working frequencies (10.5–21.5 GHz) as Equations (2), (3), (5) and (6) calculated. Similarly, in the second row, after the phase profile of the topological charge *l* = −1 was mixed with the phase sequences with a period of Γ_2_ = 64 mm along the −*x* and +*y* axes, the vortex beam skewed to azimuth *φ*_r2_ = 135°, and the frequency-controlled scanning elevation *θ*_r2_ range was 17.9°–39.1°.

Furthermore, the addition theorem in complex form combines two different phase profiles of e^jΦ^ with different functions, and the added pattern directly motivates the two functions simultaneously without any perturbations [38]:

(7) $$e^{j\Phi_{1}\left(x,y\right)}\cdot e^{j\Phi_{2}\left(x,y\right)}=e^{j\Phi_{0}\left(x,y\right)}$$

where e^jΦ^^0^^(x,y)^ is the phase distribution on the multifunction metasurface after a complex addition operation. According to the superposed phase pattern in Figure 4k, the metallic patch rotating angles α_r_ were thereupon calculated, as shown in Figure 4l, and a 32 × 32 metasurface was constructed by the wideband PB meta-atom. Under the irradiation of the CP plane wave, it concurrently steered two asymmetric OAM vortex beams to separate directions with different modes, which continuously scanned in different angles following the frequency variation.

## 3. Results and Discussion

### 3.1. Simulation Results

**Figure 5:**
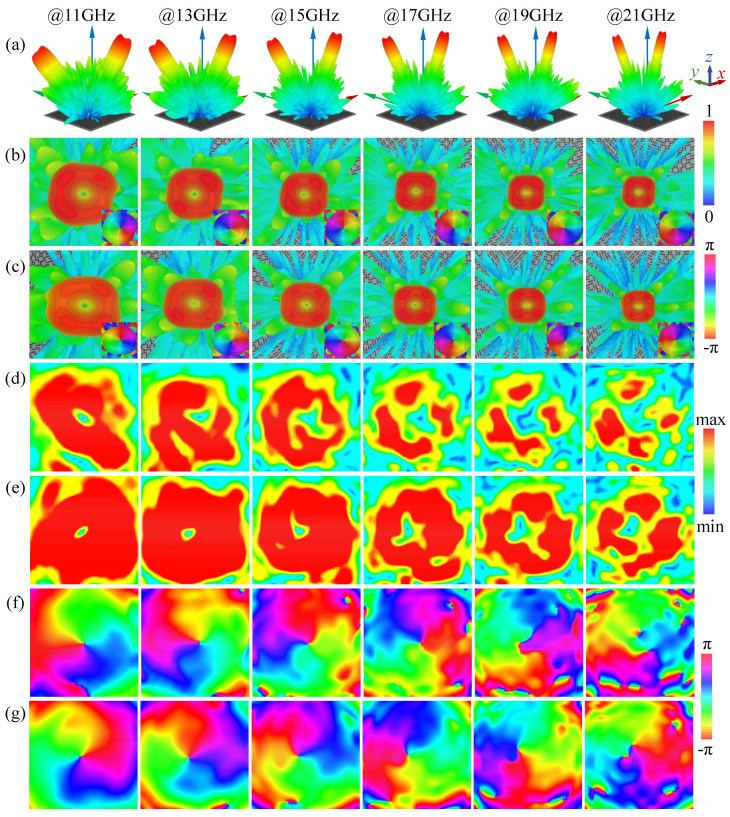
Simulated results at different frequencies. The far-field normalized 3D scattering pattern and corresponding phase in (**a**) upper half space, (**b**) fourth quadrant space, and (**c**) second quadrant space of the metasurface. The near-field amplitude of vortex wave with OAM mode (**d**) *l* = 1 in fourth quadrant space and (**e**) *l* = −1 in second quadrant space. The near-field phase of vortex wave with OAM mode (**f**) *l* = 1 in fourth quadrant space and (**g**) *l* = −1 in second quadrant space.

From the frequency range of 10.5 to 21.5 GHz, the full-wave simulations were carried out by CST Studio Suite to detect the generation of frequency-controlled dual-mode vortex waves. Figure 5 shows the far- and near-field simulated results at 11, 13, 15, 17, 19, and 21 GHz under RHCP plane wave incidence.

Figure 5a shows the normalized 3D far-field scattering pattern of the co-polarization component. As the frequency increased, the asymmetric vortex dual beams were steered to different directions and gradually approached the normal in the *φ* = 315° (135°) plane, while the divergence angle of the beams decreased, which is exactly the result predicted by phase calculation. In addition, as shown in Figure 5b,c, the hollow amplitude and ringed intensity distribution were aimed at their respective directions at different frequency points. The 2π phase profiles with opposite helixes were obtained from the two beams, revealing that the OAM waves had topological charges of *l* = 1 and −1 in the fourth and second quadrant spaces, respectively.

Meanwhile, twelve 300 × 300 mm^2^ near-field observation planes were 500 mm away from the metasurface center and perpendicular to the beam directions at six frequency points. The sampled electric field component showed a donut-like amplitude at the central area across the wideband in Figure 5d,e. Clear spiral phase distributions with OAM orders of 1 and −1 at all frequencies can be observed in Figure 5f,g. At high frequencies, the incomplete ring intensity was due to the small beam deviation causing the reflected cross-polarization component in the normal to affect the OAM wave of the RHCP. Consequently, the gradually variational intensity null and phase singularity kept in line with the far-field pattern at different frequencies, which showed the generation of the asymmetric OAM-carrying vortex EM waves.

### 3.2. Experiment Results

**Figure 6:**
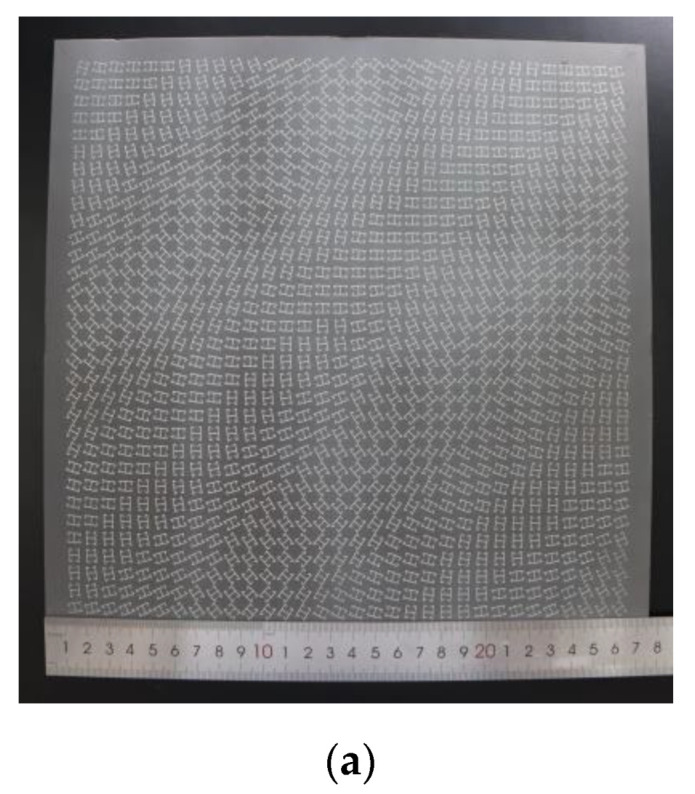

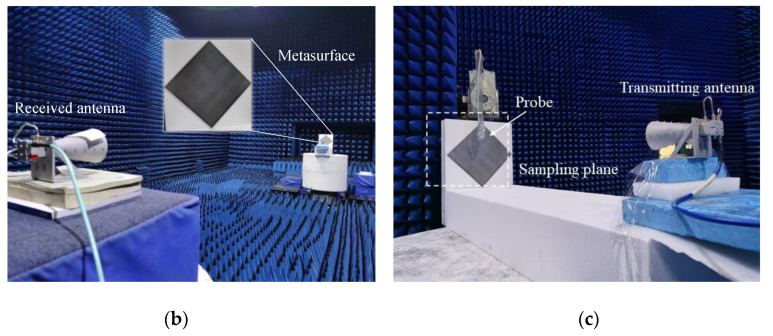
Photograph and testing environment. (**a**) The photograph of the manufactured metasurface. (**b**) Far-field testing environment. (**c**) Near-field testing environment.

To verify the intended performance of the metasurface, a prototype was fabricated using printed circuit board (PCB) technology, which is displayed in Figure 6a. The measurement was carried out in a microwave anechoic chamber, while the prototype and a circularly polarized horn antenna (HD-80180, 8–18 GHz) were fixed on a foam turntable with a distance of 1500 mm. Due to the bandwidth limitation of the transmitting antenna, the vector network analyzer (Anritsu MS4644A) recorded the far-field scattering pattern from 10.5–18 GHz. For the near-field test, the sample was rotated so that the RF coaxial cables could scan a plane perpendicular to the beams. The far- and near-field testing environments are shown in Figure 6b,c.

**Figure 7:**
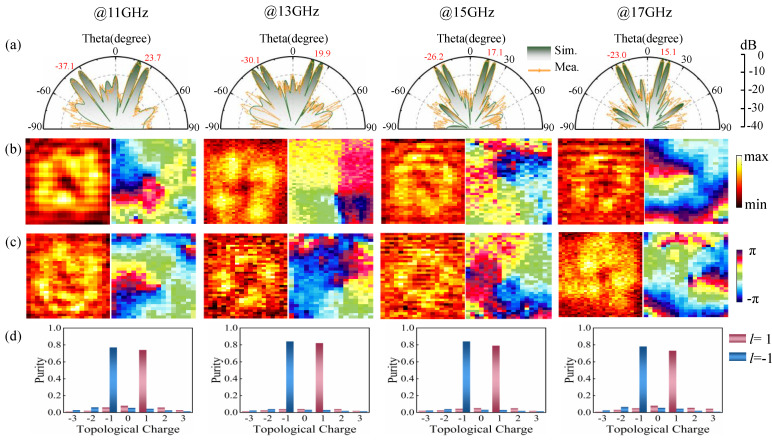
Experimental results at different frequencies. (**a**) Far-field normalized 1D scattering pattern at a *φ* = 315° plane. Near-field amplitude and phase of vortex wave with OAM mode (**b**) *l* = 1 in fourth quadrant space and (**c**) *l* = −1 in second quadrant space. (**d**) The purity of the OAM mode with different topological charges.

As can be seen from the 1D pattern of *φ* = 315° in Figure 7a, the features of the beam hollow outline in the experimental results were in agreement with the simulation. At each frequency point, the two beams pointed exactly at the precalculated angle, with acceptable side lobes. Moreover, after the decomposition of the electric field, the near-field amplitude and phase characteristics of the extracted RHCP displayed in Figure 7b,c matched the former. The distinct energy fall and phase profile, winding by ±2π in the central zone of the electric fields, appeared as expected in the operating band, verifying the generation of vortex waves carrying OAM modes of *l* = 1 and −1. The non-uniform color lump of the near-field result was related to the difference in the sampling distance. In this test, errors may have been caused by the mounting alignment, manufacturing process, and nonideal excitation source.

In addition, for the quantitative analysis of OAM waves, with the phase singularity of the vortex beam as the center of the circle, its mode decomposition was implemented with the Fourier transform as follows [42]:

(8) $$A_{l}=\frac{1}{2\pi }\int_{0}^{2\pi }\psi \left(\varphi \right)e^{-jl\varphi }d\varphi$$

(9) $$\psi \left(\varphi \right)=\sum_{l}A_{l}e^{jl\varphi }$$

in which *Ψ*(*φ*) is the RHCP component electric field on the circumference with the beam direction as the axis, and A*_l_* denotes the spectrum weight corresponding to each topological charge. Here, the OAM modes from *l* = −3 to 3 are advised, and the mode purity of the OAM mode *l* is interpreted as

(10) $$Purity=\frac{A_{l}^{2}}{\sum_{l^{\prime }=-3}^{3}A_{l^{\prime }}^{2}}$$

As depicted in Figure 7d, the experimentally measured mode purity of *l* = 1 and −1 at different frequencies in the second and fourth quadrant spaces were both around 80%, which demonstrated the effectiveness of the dual-mode OAM beams. The mode purity value for OAM mode *l* = 1 was less than that for mode *l* = −1, which was due to the energy of the RHCP vortex wave near the normal direction being dispersed by the reflected cross-polarization wave.

## 4. Conclusions

An efficient broadband PB metasurface element is proposed, which can achieve a high co-polarized reflection amplitude and a continuous phase gradient of 0–2π under the illumination of CP plane waves. According to the phase design, metasurface-reflecting dual-mode asymmetric dual-OAM waves were aligned. Under the continuous modulation of frequency (10.5–21.5 GHz), the OAM waves with the mode of 1 and −1 were motivated in *φ* = 315° and 135° planes, scanning at the angles of 11.9°–24.9° and 17.9°–39.1°, respectively. The feasibility of generating frequency-controlled asymmetric dual beams and the validity of dual-mode OAM characteristics were confirmed by simulated and experimental results of the near and far fields. This is likely the first proposed design strategy using a passive metasurface to generate a frequency scanning dual-mode dual-OAM wave. On the one hand, associated with the leaky wave metasurface, it can vary the period length to improve the frequency scanning efficiency and facilitate information transmission. On the other hand, the addition operation of complex phases has great potential for expanding passive metasurface functionality and boosting channel multiplexing capabilities.
